# Preeclampsia Is a Double-Hit Vascular Disorder: The VEGF-HO-1-CSE Axis

**DOI:** 10.3390/biom16030436

**Published:** 2026-03-13

**Authors:** Asif Ahmed, Stephen K. Smith, Shakil Ahmad, Keqing Wang

**Affiliations:** 1 Mirzyme Therapeutics Limited, Innovation Birmingham Campus, Faraday Wharf, Holt Street, Birmingham B7 4BB, UK; stephen.smith@mirzyme.com; 2 Faculty of Environmental and Life Sciences, School of Health Sciences, President’s Office, University of Southampton, Southampton SO17 1BJ, UK; 3 Aston Medical Research Institute, Aston Medical School, Aston University, Birmingham B4 7ET, UK; s.ahmad@aston.ac.uk (S.A.); k.wang@aston.ac.uk (K.W.)

**Keywords:** preeclampsia, angiogenic imbalance, soluble Flt-1 (sFlt-1), soluble endoglin (sEng), heme oxygenase-1 (HO-1), biliverdin reductase (BVR), hydrogen sulfide (H2S), cystathionine γ-lyase (CSE), VEGF, PlGF

## Abstract

Preeclampsia is a double-hit vascular disorder centred on the VEGF-HO-1-CSE axis. First, excess placental soluble Flt-1 (sFlt-1) neutralises vascular endothelial growth factor (VEGF) and placental growth factor (PlGF), producing an angiogenic deficit that drives endothelial dysfunction, hypertension, proteinuria and end organ injury. Second, the failure of endogenous vascular brakes, heme oxygenase-1 (HO-1/CO) and cystathionine-γ-lyase (CSE)/hydrogen sulfide (H_2_S) removes physiological restraint on anti-angiogenic factor release (sFlt-1; soluble endoglin) and amplifies oxidative–inflammatory stress, lowering the threshold at which VEGF loss precipitates severe disease. We synthesise human, animal and translational data that (i) establish placental sFlt-1 source and release, (ii) demonstrate human mechanistic causality via sFlt-1 removal, (iii) show prospective clinical validation that sFlt-1 rises and free PlGF falls before disease onset, and (iv) identify HO-1 and CSE/H_2_S as protective pathways that restrain anti-angiogenic drive. Finally, we summarise preclinical evidence that the orally administered H_2_S-donor prodrug MZe786 restores the HO-1/CSE axis, lowers sFlt-1 and soluble endoglin (sEng), and improves maternal haemodynamics and foetal outcomes across complementary pregnancy models, and we outline the role of sFlt-1/PlGF and M-PREG-based triage in clinical decision making. While valuable for short-term triage, current sFlt-1/PlGF-based approaches cannot sub-stratify among positive cases. Framing severe preeclampsia as a double-hit vascular disorder provides a biologically grounded framework that can inform risk stratification strategies like M-PREG^®^, a clinical decision support system informed by the double hit framework, and prevention strategies, pairing early risk stratification with mechanism-informed interventions.

## 1. Epidemiology and Clinical Burden

Preeclampsia complicates 5–8% of pregnancies worldwide and remains a leading cause of maternal and perinatal morbidity and mortality. The burden is not evenly shared. In low- and middle-income countries, maternal death rates are many times higher than in wealthy nations due to limited access to antenatal and emergency care. Significant disparities persist even in high-resource settings; for example, in the United States, cause-specific mortality from preeclampsia/eclampsia is approximately five-fold higher among non-Hispanic Black women compared to non-Hispanic White women [1].

The clinical and human cost is staggering. Globally, preeclampsia is estimated to claim the lives of 76,000 mothers and 500,000 babies annually, and it leaves many more survivors with lasting cardiovascular, renal, and neurological sequelae [2]. The only definitive cure remains delivery of the placenta and baby, often prematurely, which creates serious challenges for newborn care.

Preeclampsia is characterised by new-onset hypertension after 20 weeks’ gestation with proteinuria and/or maternal organ dysfunction, and is frequently associated with foetal growth restriction. If not recognised and treated, it can progress rapidly to eclampsia, a life-threatening convulsive state marked by seizures, stroke, and death. The delivery of the placenta remains the only definitive cure.

This clinical syndrome, whose pathogenesis has eluded medicine for decades, underscores an urgent, unmet need. While timely diagnosis and delivery mitigate mortality in high-resource settings, the disease itself remains unprevented. In low- and middle-income countries, where these safeguards are often absent, preeclampsia persists as a leading cause of maternal and perinatal death. This global burden, combined with the syndrome’s potential for rapid onset even in previously low-risk pregnancies, highlights the critical necessity for reliable early risk stratification and mechanism-informed prevention strategies.

Research over three decades has identified multiple interacting pathophysiological processes that contribute to preeclampsia, including defective trophoblast invasion and spiral artery remodelling [3,4], complement dysregulation and aberrant innate immune activation [5,6], immune maladaptation and placental stress responses with systemic inflammatory features [7,8], and the activation of the renin–angiotensin system with agonistic autoantibodies directed against the angiotensin II type 1 receptor [9,10] and mechanistic vascular effects described [11,12]. These frameworks are not mutually exclusive. Rather, they likely represent overlapping upstream triggers and downstream amplifiers that converge on a common final pathway of maternal endothelial dysfunction. In this context, angiogenic imbalance and the failure of endogenous vascular cytoprotective buffering provide a mechanistically testable axis through which diverse upstream events may lead to systemic vascular injury. This review focuses on the angiogenic imbalance axis, specifically the interplay between the placental release of excess soluble Flt-1 (sFlt-1) and the failure of endogenous cytoprotective pathways centred on heme oxygenase-1 (HO-1) and cystathionine γ-lyase (CSE)/hydrogen sulfide (H_2_S), for which the translational evidence is the most mature and the therapeutic implications are most clearly defined.

## 2. The Angiogenic Imbalance Model: From Hypothesis to Validation (Hit 1)

### 2.1. Placental VEGF Signalling

A conceptual shift in the mid-1990s proposed that preeclampsia arose not from primary hypertension, but from a loss of vascular endothelial growth factor (VEGF) bioactivity within the maternal vasculature [13]. This was grounded in foundational studies demonstrating that the human placenta expresses VEGF, but that it also expresses the full-length VEGF receptor-1 and the fms-like tyrosine kinase receptor (Flt-1) [14]. Placental growth factor (PlGF) is another ligand for the VEGF receptor-1 that is expressed exclusively during pregnancy, thus establishing it as a tissue capable of autocrine and paracrine angiogenic signalling [14,15,16,17].

In 1997, this body of placental molecular evidence was synthesised into an explicit “VEGF loss” hypothesis, proposing that preeclampsia results from the neutralisation of VEGF, plausibly by a soluble form of its receptor, sFlt-1 [13]. In 1998, Clark et al. identified a truncated version of Flt-1 that lacks the transmembrane and cytoplasmic domains of the full-length tyrosine kinase receptor Flt-1/VEGF receptor-1 and demonstrated that human placental villous explants release a soluble VEGF receptor consistent with sFlt-1 into conditioned media and the maternal circulation, establishing the placenta as a source of circulating VEGF antagonism [18]. This circulating receptor binds free VEGF, thereby providing the key molecular mediator predicted by the VEGF loss hypothesis.

**Figure 1:**
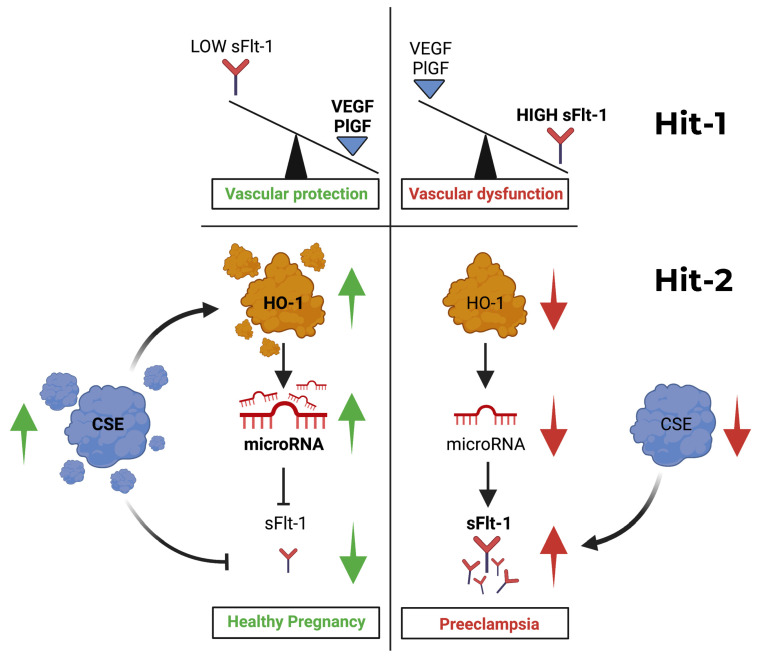
The double-hit vascular disorder model of preeclampsia. Hit 1 (Angiogenic Deficit): Excess placental sFlt-1 neutralises VEGF and PlGF, driving maternal endothelial dysfunction. Hit 2 (Failed Brakes): The failure of the endogenous cytoprotective pathways HO-1/CO and CSE/H_2_S amplifies sFlt-1/sEng release and oxidative stress, lowering the threshold for clinical disease. **Left panel**—Healthy pregnancy: Under normal conditions, the cystathionine γ-lyase/hydrogen sulfide (CSE/H_2_S) pathway modulates heme oxygenase-1 (HO-1) activity, maintaining high levels of specific microRNAs that bind to the 3′-UTR of FLT1 mRNA. This post-transcriptional regulation suppresses placental soluble Flt-1 (sFlt-1) release. Consequently, the circulating levels of free vascular endothelial growth factor (VEGF) and placental growth factor (PlGF) remain high, supporting normal endothelial function, vascular homeostasis, and foetal-placental development. Hit 1 is absent. **Right panel**—Preeclampsia: When HO-1 and/or CSE/H_2_S pathways are compromised (Hit 2: failure of cytoprotective brakes), the microRNA-mediated brake on FLT1 mRNA is lifted. This leads to unchecked placental production and release of sFlt-1 into the maternal circulation (Hit 1: angiogenic deficit). Excess sFlt-1 neutralises circulating VEGF and PlGF, resulting in decreased bioavailable pro-angiogenic factors. This imbalance leads to maternal endothelial dysfunction, hypertension, oedema, proteinurea, systemic oxidative stress, and end organ injury, culminating in the clinical syndrome of preeclampsia. Key to symbols: Green arrows indicate activation or production. Red arrows indicate inhibition, suppression or impaired signalling. The model integrates key evidence: human placental causality via sFlt-1 removal [26], prospective biomarker validation [23,27,28] and the therapeutic rationale for restoring protective brakes [29,30,31,32].

Importantly, these early functional studies demonstrated that disease severity reflected the relative imbalance between anti-angiogenic and pro-angiogenic signals, rather than being the consequence of absolute concentrations alone (Figure 1). Early studies demonstrated that increasing placental sFlt-1 release, possibly driven by hypoxia and VEGF itself, impaired endothelial migration, with the most severe angiogenic inhibition observed in preeclampsia compared with foetal growth restriction. Expressed as a PlGF/sFlt-1 relationship, lower values were associated with worse biological outcomes, anticipating later ratio-based approaches [19].

### 2.2. Hypoxia, HIF Signalling, and sFlt-1 Regulation

Placental hypoxia is a recognised upstream stressor in early-onset and severe preeclampsia. Hypoxia stabilises hypoxia-inducible factors (HIFs) in trophoblasts, enabling the transcriptional activation of target genes through binding to functional hypoxia response elements (HREs) within regulatory regions of both *VEGFA* and *FLT1* [20]. Hypoxic stimulation increases the transcription of *FLT1* and its soluble splice variants in placental trophoblasts [21]. Importantly, experimental studies demonstrate that HIF-2α, rather than HIF-1α, is the dominant mediator of hypoxia-induced sFlt-1 upregulation in trophoblasts; silencing HIF-2α attenuates hypoxia-driven sFlt-1 expression and secretion [21].

Although hypoxia can increase local placental VEGF expression, it simultaneously enhances sFlt-1 production and its release into the maternal circulation [22]. The preferential secretion of soluble Flt-1 shifts the systemic angiogenic balance toward VEGF neutralisation, resulting in reduced circulating free VEGF bioactivity despite increased local transcription [23,24]. This resolves the apparent paradox whereby hypoxia induces VEGF gene expression while maternal serum exhibits an angiogenic deficit. The key milestones preceding and supporting this hypothesis are summarised in Table 1.

Beyond transcriptional control, cell-type-specific post-transcriptional mechanisms further regulate sFlt-1 production. In non-placental endothelial cells, the alternative polyadenylation of *FLT1* pre-mRNA is influenced by heterogeneous nuclear ribonucleoprotein D (hnRNP D) and arginine methylation status, modulating soluble Flt-1 production independently of hypoxia signalling [25]. These findings highlight that the regulation of sFlt-1 is multi-layered and context dependent, with trophoblast hypoxia–HIF signalling playing a central role in the placental compartment relevant to preeclampsia.

The systemic administration of sFlt-1 to pregnant rats induces hypertension, proteinuria, and glomerular endotheliosis, changes that can be attenuated by VEGF supplementation, thereby supporting maternal causality in vivo [24]. Complementing this animal work, direct human tissue evidence that sFlt-1 mediates angiogenic suppression was demonstrated in 2004, when the selective immunodepletion of sFlt-1 from preeclamptic placental conditioned media restored angiogenesis ex vivo [26]. This provided direct human tissue evidence that excess placental sFlt-1 is functionally responsible for angiogenic suppression [26] and together these observations provided a mechanistic rationale for translational approaches aimed at lowering circulating sFlt-1 in severe disease [33,34].

### 2.3. Soluble Endoglin Strengthens the Anti-Angiogenic State

A second placenta derived anti-angiogenic factor, soluble endoglin (sEng), strengthened the angiogenic imbalance model by explaining why some women deteriorate rapidly with severe maternal end organ injury. Endoglin is a co-receptor for TGF-β family signalling in the vasculature. The soluble form of endoglin antagonises pro-angiogenic and vasculoprotective signalling, compounding endothelial dysfunction [35]. Clinically, higher circulating sEng is associated with the severity of preeclampsia and with adverse outcomes, and biologically it acts synergistically with sFlt-1 to drive a more toxic anti-angiogenic state [35,36].

Soluble Flt-1 neutralises VEGF and PlGF, whereas sEng perturbs complementary endothelial signalling pathways. Together, elevations in both mediators lower the threshold for vascular decompensation. This suggests that the most severe phenotype reflects not simply an isolated rise in sFlt-1, but a combined anti-angiogenic burden, which amplifies endothelial injury. The rationale for targeting HO-1/CSE-linked cytoprotective signalling is therefore to restore broader endothelial resilience rather than solely reducing circulating sFlt-1 levels. Preeclampsia therefore arises not simply from elevated sFlt-1 levels, but from the loss of endogenous cytoprotective restraint that normally limits placental release of anti-angiogenic factors.

**Table 1:** Timeline of key discoveries and translational mapping.

Year	Discovery	Explanation	References
1993	VEGF mRNA expression and localisation in human placenta.	VEGF mRNA localised to first trimester villous trophoblast, term extravillous trophoblast, foetal Hofbauer cells and maternal decidual cells, supporting placental and decidual VEGF expression during pregnancy.	[15]
1994	Discovery of VEGF receptor-1 (Flt-1) in placental trophoblast.	Molecular evidence that placental trophoblast expresses VEGF receptor 1 (Flt-1), indicating the capacity for VEGF-responsive signalling within placental tissue.	[14]
1995	VEGF–Flt-1 co-localisation in human placenta.	Co-localisation of VEGF protein and Flt-1 receptor in trophoblast, decidua and Hofbauer cells, supporting autocrine and paracrine VEGF signalling within the placenta.	[16]
1996	PlGF localisation in human placenta.	PlGF mRNA and protein localised in term human placenta with methodological detail and images, establishing PlGF as a placental ligand relevant to angiogenic balance.	[17]
1997	Loss of VEGF activity hypothesis in preeclampsia.	Proposed that preeclampsia arises from loss of VEGF bioactivity, plausibly mediated by endogenous soluble Flt-1. First explicit mechanistic hypothesis centred on VEGF antagonism.	[13]
1998	Human placenta produces and releases soluble VEGF receptor-1 (sFlt-1).	sFlt-1 mRNA demonstrated in trophoblast, villous explants release sFlt-1, and maternal serum contains a VEGF-binding protein consistent with sFlt-1, establishing placental source and release into the maternal circulation.	[18]
2000	Heme oxygenase-1 (HO-1) protective pathway in human placenta (first direct evidence).	HO-1 induction attenuates TNF-α-mediated cytotoxicity in human placental villous explants and causes carbon monoxide-dependent vasorelaxation; HO-1 protein is reduced in preeclamptic placenta, supporting HO-1 as an endogenous placental cytoprotective pathway in pregnancy.	[37]
2001	Functional imbalance (PlGF to sFlt-1 relationship).	Early evidence that lower PlGF to sFlt-1 relationship associates with worse biological outcomes, anticipating later ratio-based approaches.	[19]
2003	In vivo maternal causality (pregnant rats).	Systemic sFlt-1 in pregnant rats induces hypertension, proteinuria and glomerular endotheliosis; reduced free VEGF and PlGF and endothelial dysfunction are reversible with VEGF and PlGF.	[24]
2004	Human mechanistic causality.	Selective removal of sFlt-1 from preeclamptic placental conditioned medium restores angiogenesis ex vivo, providing direct human tissue evidence for sFlt-1 mediated angiogenic suppression. Direct human tissue proof of causality.	[26]
2004	Early clinical validation.	Rising sFlt-1 and falling free PlGF precede clinical preeclampsia, track severity, and fall postpartum, providing clinical evidence consistent with the angiogenic imbalance mechanism. Provided the first large clinical evidence for the angiogenic imbalance theory.	[23,27,28]
2006	Soluble endoglin (sEng) enters the preeclampsia model.	Circulating sEng is elevated before preeclampsia and, together with sFlt-1, intensifies endothelial dysfunction; the co-administration of sEng and sFlt-1 produces a more severe preeclampsia-like phenotype in pregnant animals, supporting synergistic anti-angiogenic drive. sFlt-1 plus sEng is a “high-toxicity” combination.	[35,36]
2007	HO-1/CO protective brake.	Upregulation of HO-1 reduces sFlt-1 and sEng release; HO-1 deficiency increases both, identifying HO-1 as an endogenous pathway restraining anti-angiogenic factor release.	[29]
2013	CSE/(H_2_S) protective brake.	CSE is reduced in preeclampsia; CSE inhibition increases sFlt-1 and sEng, whereas an H_2_S donor reduces anti-angiogenic factors and improves foetal growth in mice.	[30]
2016	Clinical triage adoption (PROGNOSIS).	Multicentre validation of the sFlt-1 to PlGF ratio; a cut-off ≤38 reliably rules out preeclampsia within 1 week in women with suspected disease.	[38]
2019	Real world effectiveness and implementation: PARROT trial.	Subsequent analyses extended rule out to up to 4 weeks and informed retesting strategies. A randomised trial of PlGF-based testing in UK maternity units showed a shorter time to diagnosis and reduced severe maternal adverse outcomes.	[39,40]
2020–2021	Oral small molecule therapy development (MZe786).	In refined mouse RUPP models, MZe786 lowers sFlt-1, reduces MAP and oxidative stress, and improves foetal outcomes. In an HO-1-compromised, high-sFlt-1 pregnancy model, MZe786 reduces circulating sFlt-1 and sEng, whereas aspirin does not, with improved maternal and foetal outcomes across complementary models.	[31,32]
2025–2026	Translational/Developmental.	M-PREG^®^ is a clinical decision support system for risk stratification in suspected preeclampsia. In parallel, GMP grade manufacturing and the formulation of MZe786 have been completed, and IND submission is in preparation.	

### 2.4. Heme Oxygenase-1: A Master Cytoprotective Brake

The heme oxygenase–biliverdin reductase (HO-1/BVR) system is a critical endogenous cytoprotective and signalling axis in vascular tissues. HO-1 degrades heme to biliverdin, carbon monoxide (CO), and iron. Biliverdin is subsequently reduced to bilirubin by biliverdin reductase (BVR). The products of this pathway confer protection via antioxidant effects (through a bilirubin↔biliverdin redox cycle) and vasodilatory signalling (via CO) [41,42].

In pregnancy, this axis is indispensable. Ahmed et al. (2000) demonstrated, for the first time in human placental tissue, that HO-1 induction protects against cytokine-mediated cytotoxicity and mediates vasorelaxation, and that its expression is reduced in preeclampsia [37]. This established HO-1 as a vital guardian of placental and vascular health in pregnancy.

Within the double hit framework (Figure 1), HO-1/BVR constitutes a major maternal cytoprotective “brake” (Hit 2). Its failure amplifies anti-angiogenic factor release and oxidative–inflammatory stress, lowering the threshold at which the angiogenic deficit (Hit 1) becomes clinically catastrophic. Subsequent studies have shown that HO-1 suppresses the release of both sFlt-1 and soluble endoglin, confirming its central role as a protective brake [29].

Beyond its vasodilatory and antioxidant effects, HO-1 directly intersects with iron metabolism. Heme degradation liberates ferrous iron, which induces ferritin expression and modulates ferroportin-mediated export. Hepcidin regulates systemic iron trafficking, and the dysregulation of this axis may increase labile iron pools and lipid peroxidation. Recent evidence demonstrates that placental hypoxia induces ferroptosis, characterised by iron accumulation, glutathione depletion, reduced glutathione peroxidase 4 (GPx4) activity, and lipid peroxidation, with the extrusion of iron-rich, lipid peroxide-laden small extracellular vesicles into the maternal circulation, thereby contributing to endothelial activation and impaired angiogenesis [43]. Emerging evidence further implicates ferroptosis, an iron-dependent form of regulated cell death driven by lipid peroxidation, in pathological pregnancy states [44]. Increased oxidative stress and impaired antioxidant buffering may render endothelial cells more susceptible to ferroptotic injury under angiogenic stress conditions. In this context, the failure of HO-1-mediated cytoprotective buffering may amplify both anti-angiogenic signalling and iron-dependent oxidative injuries, integrating redox and angiogenic pathways within the double hit framework.

In the years that followed, independent studies confirmed and extended this model. Large prospective studies demonstrated that alterations in circulating PlGF and sFlt-1 precede clinical disease and can predict risk weeks before symptom onset [23,27]. Independent longitudinal studies further showed that circulating sFlt-1 rises weeks before the onset of clinical preeclampsia and correlates with disease severity [28].

The angiogenic imbalance paradigm has substantially influenced mechanistic and translational research directions in preeclampsia over the past two decades. The significance of this conceptual shift lies in its ability to integrate diverse mechanistic observations into a coherent and testable vascular framework with direct implications for improving maternal and perinatal outcomes.

### 2.5. Nitric Oxide Bioavailability as a Convergent Downstream Effector

Nitric oxide (NO) bioavailability represents the critical downstream convergence point at which both hits of the double hit model produce their final common vascular injury [45]. The functional link between VEGF and NO in placental biology was established in 1997, when it was demonstrated that human trophoblast and endothelial cells express functional Flt-1 receptors that, upon VEGF stimulation, trigger the calcium-dependent activation of constitutive NO synthase and the release of NO: this was the first demonstration that VEGF signals via Flt-1 generate NO in placental cells [46]. Subsequently, it was shown directly that addition of soluble VEGFR-1 (sFlt-1) to endothelial cells competitively antagonises VEGF-mediated Flt-1 signalling and suppresses NO production [47], providing early mechanistic evidence that the angiogenic deficit of Hit 1 operates, at least in part, by impairing NO bioavailability in the maternal vasculature. The cytoprotective pathways of Hit 2 sustain this same NO axis from a complementary direction. Carbon monoxide generated by HO-1 prevents TNF-α-induced eNOS downregulation by inhibiting NF-κB-responsive miR-155-5p biogenesis [48], while CSE-derived H_2_S directly controls endothelial NO bioavailability, as demonstrated in CSE-deficient models [49]. In preeclampsia, both axes fail simultaneously. Oxidative stress promotes eNOS uncoupling, switching the enzyme from a NO-generating to a superoxide-generating state; BH4 oxidation, L-arginine depletion by elevated placental arginase activity, and direct eNOS modification by lipid peroxidation-derived aldehydes each contribute to this transition [50]. Circulating eNOS protein concentrations are significantly lower in preeclamptic compared with normotensive pregnant women across two independent cohorts [51]. Together, these mechanisms position NO deficiency as the shared effector pathway through which angiogenic deficit (Hit 1) and failed cytoprotective brakes (Hit 2) converge to produce maternal endothelial decompensation: the two hits do not act in parallel but collapse onto the same final molecular target.

### 2.6. Aspirin Prophylaxis: Mechanistic Alignment and Limitations

Low-dose aspirin suppresses placental COX-2-derived thromboxane A_2_, restores prostacyclin/thromboxane balance, and has been shown in preeclampsia organoid models to activate PI3K-AKT-mTOR signalling and optimise mitochondrial function [52]. These actions map onto the angiogenic arm of Hit 1, and the double hit model provides a mechanistic framework that retrospectively explains both aspirin’s partial clinical activity and its limitations. In the ASPRE trial, the early initiation of 150 mg aspirin before 16 weeks in first-trimester screen-positive women reduced preterm preeclampsia by approximately 62% in a highly selected, high-adherence cohort [53]. Across broader meta-analytic evidence, however, the absolute risk reduction for preeclampsia overall is modest, at 2 to 5% in high-risk populations [54,55,56,57] and the clinical benefit is highly dependent on gestation of initiation, dose, and adherence. Most recently, the CASPER trial, a double-blinded cluster RCT in high-risk Malawian women all receiving calcium supplementation, found no statistically significant reduction in preeclampsia with 150 mg aspirin (adjusted OR 1.16, 95% CI 0.41–3.41), with an adherence of 69% and a predominance of chronic hypertension; the authors concluded that aspirin alone is insufficient where established maternal vascular disease is the dominant driver [58]. This conclusion aligns with our own preclinical findings, in which aspirin did not suppress sFlt-1 in preeclampsia animal models, whereas MZe786 did [31,32]. These converging lines of evidence suggest that the correction of the sFlt-1-mediated angiogenic deficit requires the engagement of the cytoprotective axis, not COX inhibition alone. MZe786 was designed precisely for this purpose: its molecular backbone retains the COX-inhibitory and prostacyclin-restoring properties of aspirin, while the appended H_2_S-releasing moiety simultaneously addresses both arms of the double hit model, augmenting HO-1 and CSE cytoprotective signalling and thereby targeting the failure of Hit 2 that aspirin monotherapy leaves entirely unaddressed. This dual-mechanism architecture is mechanistically grounded in the double hit framework, though the therapeutic advantage of MZe786 over aspirin monotherapy remains a hypothesis supported by preclinical data and awaits clinical validation.

### 2.7. Conceptual Evolution: From Protective Pathways to the Double Hit Model

This framework, in which the failure of endogenous vascular protective systems determines disease severity, evolves from the accelerator–brake hypothesis first formally proposed to explain preeclampsia pathophysiology [59]. It was proposed that “the pathogenesis of preeclampsia is largely due to loss of HO activity,” resulting in excessive anti-angiogenic factor release [59]. This built upon the observation that the incidence of preeclampsia is reduced in smokers, a paradox linked to carbon monoxide (CO) from cigarette smoke suppressing placental sFlt-1 release. This provided an early epidemiological clue that augmenting the HO-1/CO ‘brake’ could modify disease risk.

This concept was refined using the Bradford Hill criteria for causation [60]. It argued that the angiogenic imbalance model demonstrated strength, consistency, temporality, and biological plausibility, while complementary frameworks (e.g., defective spiral artery remodelling and systemic inflammation) provide important biological context and may represent downstream amplifiers. The subsequent identification of the CSE/H_2_S pathway as a second protective brake [30] completed the core circuitry of the endogenous cytoprotective system.

The synthesis presented here refines this into the testable ‘double-hit vascular disorder’ model. Hit 1 (angiogenic deficit) defines the necessary insult. Hit 2 (failed cytoprotective brakes) explains the variable penetrance and severity, providing a mechanistic basis for why only some pregnancies with placental stress progress to clinical disease. This model moves beyond association to provide a unified, causative framework that explains clinical heterogeneity, underpins biomarker utility, and directly informs therapeutic strategy.

## 3. Failure of Cytoprotective Brakes Amplifies Disease (Hit 2)

### 3.1. Cytoprotective Pathways: HO-1/CO and CSE/H_2_S

Identifying the trigger for preeclampsia was only part of the puzzle. The next question was simple: what natural systems normally keep the condition under control, and why do they fail?

Subsequent work identified a parallel cytoprotective system that also acts like a safety brake. The dysregulation of the cystathionine γ-lyase/hydrogen sulfide (CSE/H_2_S) pathway also contributes to maternal hypertension and placental abnormalities, establishing a second protective brake that fails in preeclampsia [30].

Mechanistically, the HO-1/CO and CSE/H_2_S systems exert protective effects at multiple levels. The induction of HO-1 suppresses the placental release of sFlt-1 and soluble endoglin through carbon monoxide-dependent signalling and the modulation of redox-sensitive transcriptional pathways [29]. Carbon monoxide enhances endothelial nitric oxide bioavailability and limits inflammatory activation, thereby stabilising endothelial function under conditions of angiogenic stress. In parallel, bilirubin generated via the HO-1/BVR axis acts as a potent lipid-phase antioxidant, reducing oxidative amplification of anti-angiogenic signalling.

The CSE/H_2_S pathway provides complementary cytoprotection. Hydrogen sulfide promotes vasodilation, preserves mitochondrial bioenergetics, and suppresses reactive oxygen species accumulation. The experimental inhibition of CSE increases placental sFlt-1 and sEng release, whereas H_2_S donors restore angiogenic balance and improve vascular function in pregnancy models [30]. At the endothelial level, H_2_S supports nitric oxide signalling and enhances resistance to VEGF withdrawal-induced dysfunction.

Within the double hit framework, the failure of these cytoprotective systems lowers the threshold at which placental anti-angiogenic stress translates into systemic endothelial decompensation. Hit 2 therefore amplifies the biological consequences of Hit 1 rather than initiating placental factor release itself. This mechanistic interplay is illustrated in Figure 1.

### 3.2. HO-1 Promoter Activity and Genetic Vulnerability

Functional variation within the HMOX1 promoter refines the “protective brake” concept. HO-1 inducibility is strongly influenced by polymorphic elements within its promoter, most notably the length of a (GT)n microsatellite repeat, where longer repeat variants are associated with reduced transcriptional responsiveness and diminished HO-1 induction under stress [45,61].

In human pregnancy, long (GT)n promoter variants have been associated with susceptibility to late-onset and non-severe preeclampsia in the FINNPEC cohort, consistent with a model in which impaired HO-1 stress responsiveness lowers vascular resilience rather than acting as a primary trigger. This fits squarely within Hit 2 of the double-hit vascular disorder framework (Figure 1): once placental anti-angiogenic stress emerges, a weakened HO-1 brake permits greater endothelial injury and amplifies disease expression [45].

Prospective cohort studies have reported differences in circulating angiogenic markers across ancestral groups [62,63,64]. Among Black women, higher plasma sFlt-1 levels have been associated with markers of cardiovascular dysfunction during pregnancy and postpartum, independent of overt preeclampsia [62]. In large, diverse cohorts, Black or African American participants have been observed to exhibit higher median sFlt-1/PlGF ratios and distinct angiogenic biomarker distributions compared with White women at comparable gestational windows [63,64]. These findings suggest that baseline angiogenic set points may vary across populations and could influence both susceptibility to placental stress and the performance of biomarker-based risk stratification strategies.

Within the context of our brake theory, the ancestral enrichment of HMOX1 promoter variants linked to lower HO-1 inducibility provides a plausible biological substrate for reduced cytoprotective reserve, amplifying the impact of the anti-angiogenic load once it emerges [45,65]. Together, these data support a mechanistic second hit in which constrained HO-1 inducibility and a shifted angiogenic equilibrium lower the threshold for maternal vascular decompensation when placental anti-angiogenic factors rise.

### 3.3. From Mechanism to Medicine: Translational Implications of Restoring the HO-1/CSE Axis

Guided by the double hit model, MZe786 was developed as an oral agent to restore activity within the HO-1/CSE cytoprotective pathway. In preclinical studies, MZe786 prevented preeclampsia-like features in pregnancies characterised by HO-1 deficiency and elevated sFlt-1, supporting the therapeutic rationale of reinstating endogenous protective mechanisms [31,32]. In the refined Reduced Uterine Perfusion Pressure (RUPP) mouse model, the oral restoration of H_2_S signalling with MZe786 lowered circulating sFlt-1 and sEng as well as sFlt-1 concentrations in amniotic fluid, improved maternal vascular function, attenuated hypertension and renal injury in dams, and reduced foetal loss [31]. These findings demonstrate that the modulation of cytoprotective signalling can influence both upstream placental anti-angiogenic output and downstream maternal–foetal pathology in vivo. The oral formulation further enhances translational potential, particularly for use in low-resource settings. Clinical trials will be required to determine safety and efficacy in human pregnancy.

Importantly, the translational logic of this framework is supported by convergence between clinical biomarker stratification and experimental pathway modulation. Prospective human studies demonstrate that circulating angiogenic imbalance, including elevated maternal plasma sFlt-1 and altered PlGF relationships, can be quantified and used to risk stratify women prior to clinical deterioration [23,27,28]. Together, these experimental and mechanistic data support the plausibility of the double hit model, linking measurable angiogenic disruption with the targeted restoration of endogenous protective pathways. Although clinical validation remains necessary, this convergence enhances the biological and translational coherence of the double hit model.

### 3.4. Validating the Axis: Recent Advances in Therapeutics and Subphenotyping

The predictive power of the double hit model is demonstrated by its success in guiding therapeutic development and refining patient stratification.

A distinct therapeutic strategy involves the augmentation of endogenous vasoactive pathways without directly modifying placental anti-angiogenic factor production. DM199 is a recombinant form of human tissue kallikrein-1 (KLK-1), a serine protease that activates the kallikrein–kinin system. KLK-1 cleaves low-molecular-weight kininogen to generate bradykinin, which signals through endothelial B2 receptors to stimulate nitric oxide and prostacyclin release, promoting vasodilation and improved microvascular perfusion [66,67]. Because increased vascular tone and endothelial dysfunction are central features of preeclampsia, the augmentation of this pathway has been proposed as a haemodynamic stabilisation strategy. The South African Phase IB/IIA study evaluating DM199 in preeclampsia and foetal growth restriction has been described in a peer-reviewed trial protocol [68] and is registered at ClinicalTrials.gov (NCT06875141) [69]. The study employs an open-label dose escalation design assessing safety, tolerability, and exploratory haemodynamic endpoints in women already scheduled for delivery. Preliminary news reports have suggested reductions in maternal blood pressure; however, the peer-reviewed controlled efficacy data, effects on circulating sFlt-1 or soluble endoglin, and evidence of meaningful gestational prolongation have not yet been reported. Larger, controlled trials will be required to determine whether haemodynamic stabilisation translates into sustained pregnancy extension and improved perinatal outcomes.

### 3.5. Therapeutic Validation Targeting the Axis

The causal logic of the model dictates that interventions lowering sFlt-1 or boosting protective pathways should be beneficial. This has been supported on multiple fronts:

• Direct sFlt-1 Removal: Building on the finding that immunodepleting of sFlt-1 restores angiogenesis ex vivo [26], dextran sulphate apheresis has been explored as a strategy to reduce circulating sFlt-1 in severe, early-onset preeclampsia, with reports of gestational prolongation providing in vivo human proof-of-concept [34]. However, because apheresis is not molecularly selective, the concomitant depletion of other circulating proteins cannot be excluded and could contribute to observed effects.
• Restoring the Protective Brakes: The oral H_2_S-donor MZe786 represents a mechanism-informed approach targeting Hit 2. In complementary preclinical models, including a genetic model of HO-1 deficiency, MZe786 consistently reduces sEng, improves maternal hemodynamics, and, critically, improves foetal growth [31]. This validates the core therapeutic strategy of brake restoration.

### 3.6. Subphenotyping and the Double-Hit Clinical Spectrum

The model explains the clinical continuum from late-onset to severe early-onset disease. Biomarker-based subphenotyping now aligns with this biology: a “placental” phenotype is characterised by profound angiogenic imbalance (severe Hit 1), often with foetal growth restriction, while a “maternal” phenotype may exhibit a more moderate imbalance but occur in individuals with underlying endothelial susceptibility (predisposition to Hit 2). This framework is the basis of the M-PREG^®^ digital diagnostic system, which uses angiogenic markers in algorithms to stratify risk weeks before the clinical onset of the syndrome.

### 3.7. Linking to Long-Term Cardiovascular Risk

The double hit model also provides a biological lens through which to view the well-established association between preeclampsia and a woman’s subsequent risk of cardiovascular disease, including later hypertension, ischaemic heart disease, stroke, and heart failure. Meta-analyses and large cohort studies consistently show that a history of preeclampsia identifies a population with higher long-term cardiometabolic risk compared with women with normotensive pregnancies [70]. One interpretation is that pregnancy functions as an early vascular “stress test”, unmasking limited endothelial resilience and cytoprotective reserve (Hit 2 capacity). In addition, the exposure to a severe anti-angiogenic milieu (Hit 1) may contribute to persistent vascular dysfunction in susceptible individuals. This framework is intended as a mechanistically testable hypothesis linking pregnancy vascular stress biology to later-life vascular risk, while recognising that shared predisposition and causal vascular injury are not mutually exclusive explanations.

Multiple therapeutic modalities are currently being explored for the treatment or prevention of preeclampsia, including small-molecule pathway modulators, transcript-targeting oligonucleotides such as small interfering RNA (siRNA), and recombinant biologic proteins. These approaches use different pharmacokinetic approaches with consequent manufacturing complexity, delivery requirements, and health system implementation considerations. Beyond the direct removal of circulating sFlt-1, strategies aimed at inhibiting its production, for example through siRNA-mediated targeting, are mechanistically rational but raise important translational considerations related to delivery and monitoring. Small molecule approaches designed to modulate upstream protective pathways, including the restoration of the HO-1/CSE axis, represent an alternative modality. Table 2 summarises key translational distinctions across therapeutic classes without implying superiority, as comparative clinical data remain limited.

## 4. From Biomarkers to Bedside Pathways: Short Term Triage and Real-World Implementation

Angiogenic biomarkers transitioned from mechanistic insight to clinical utility when prospective studies demonstrated that circulating angiogenic imbalance can precede overt clinical deterioration. The sFlt-1/PlGF ratio has been validated as a short-horizon triage aid, with a widely used cut-off (≤38) providing a high-confidence rule-out for the development of preeclampsia within one week in women with suspected disease, thereby supporting the safe de-escalation of monitoring when clinical features are non-severe and an alternative diagnosis is plausible [38]. The primary value of these tests is therefore to reduce diagnostic uncertainty over a defined near-term window, rather than to replace clinical judgement.

In routine maternity care, implementation trials embedding PlGF-based testing within predefined management pathways have reported a shorter time to diagnosis and improvements in pathway-level decision making, with reductions in a composite of severe maternal adverse outcomes, while not being powered to assess maternal mortality [40]. Collectively, these findings support the principle that biomarkers deliver clinical value when coupled to clear action thresholds and structured care pathways, rather than being used as isolated laboratory results.

However, an important limitation is that within the “positive” or high-risk range, angiogenic biomarkers alone do not consistently sub-stratify which individuals will deteriorate most rapidly or require urgent delivery, and false negative presentations remain clinically recognised. This limitation underpins the need for stratification approaches that incorporate additional biology beyond angiogenic imbalance alone.

More broadly, landscape analyses emphasise that improvements in preeclampsia detection and management are likely to require combined approaches, including biomarker testing, validated blood pressure measurement, and structured care pathways, particularly in low-resource settings where delayed recognition contributes substantially to morbidity [71]. These modalities target different points along the disease pathway and are complementary rather than competitive.

## 5. M-PREG^®^: Clinical Decision Support for Risk Stratification

Conventional angiogenic biomarker strategies quantify placental anti-angiogenic stress. However, disease expression reflects not only the magnitude of placental insult but also the maternal capacity to buffer that insult. By integrating markers reflecting both angiogenic imbalance and cytoprotective reserve within a structured computational framework, M-PREG^®^ operationalises the double hit model at the clinical interface.

M-PREG^®^ is a UKCA-marked clinical decision support system designed to integrate routinely available angiogenic biomarkers with additional laboratory features reflecting cytoprotective reserve within a transparent computational framework to support risk stratification in women with suspected preeclampsia. By incorporating markers related to both angiogenic imbalance (Hit 1) and endogenous vascular protective pathways (Hit 2), the system operationalises the double hit framework within a structured clinical algorithm.

While sFlt-1/PlGF-based triage provides validated short-horizon rule-out capability, it does not fully resolve heterogeneity among positive cases nor distinguish underlying vascular susceptibility that may influence progression and severity. Conceptually, multi-parameter approaches such as M-PREG^®^ aim to extend biomarker utility beyond binary rule-out paradigms by improving discrimination within higher-risk groups and supporting stratified care pathways.

As with all decision support systems, prospective evaluation in appropriately designed clinical studies will be required to determine the effectiveness, implementation performance, and impact on clinically meaningful maternal and perinatal outcomes.

## 6. Towards Prediction and Prevention of Preeclampsia

This mechanistic understanding has informed translational development of two investigational approaches:

1. MZe786 is an orally active drug that stimulates the CSE and H_2_S pathway, restores angiogenic balance (a healthy balance for blood vessel growth factors), and protects maternal blood vessels. In preclinical studies, it has been shown to reduce sFlt-1, improve blood vessel function, and prevent the clinical features of preeclampsia in three separate animal models.
2. M-PREG^®^ is a UKCA-marked digital medical device for Great Britain. It uses angiogenic biomarkers (including sFlt-1 and PlGF) combined with an algorithmic analysis of routinely available laboratory measures, including bilirubin as a surrogate of HO-1 pathway activity, together with selected electrolyte and liver function parameters, to aid clinicians in risk stratifying women with suspected preeclampsia and identifying those at highest risk of deterioration within a stratified care pathway. UKCA marking indicates conformity with applicable UK medical device regulatory requirements; certification is issued by a UK Approved Body (where required) and the device is registered with the MHRA.

If supported in multicentre clinical trials, these approaches could enable integrated prediction and prevention strategies in preeclampsia research, transforming care for mothers and babies worldwide. Such frameworks are essential for the development of decision support tools, stratified trials, and ultimately guideline-level change.

MZe786 demonstrates proof-of-concept disease modification in preclinical models and warrants carefully staged clinical evaluation. While animal models demonstrate experimental reversibility, causality in human disease remains inferential and requires interventional confirmation. This framework remains testable and falsifiable; its validity will ultimately depend on whether stratified, mechanism-informed interventions demonstrate benefit in prospective multicentre human randomised controlled trials.

## 7. Conclusions and Future Directions

The evidence reviewed here supports the double-hit vascular disorder model as a mechanistically grounded framework for understanding severe preeclampsia and informing stratified clinical investigation. Within this construct, an initiating placental anti-angiogenic state (Hit 1) becomes clinically consequential when endogenous vascular protective systems fail (Hit 2), providing a unifying axis that links molecular biology, placental pathology, and maternal endothelial dysfunction.

Built upon three decades of human, experimental, and translational research, this framework integrates angiogenic imbalance, cytoprotective signalling, and emerging data on hypoxia-driven iron dysregulation and ferroptosis into a coherent vascular paradigm. It explains both the utility and limitations of short-horizon angiogenic biomarker triage, provides mechanistic rationale for dual-axis risk stratification approaches such as M-PREG^®^, and supports pathway-targeted therapeutic strategies including the restoration of the HO-1/CSE axis with oral H_2_S-donor compounds such as MZe786.

Importantly, this model does not exclude alternative upstream triggers of placental stress but proposes that diverse initiating insults converge upon a common maternal endothelial vulnerability. Angiogenic biomarkers offer a measurable readout of placental stress, while indices of cytoprotective reserve may account for inter-individual differences in susceptibility and disease severity.

Future progress now depends on the rigorous prospective evaluation of stratified diagnostic tools and mechanism-informed interventions. Determining whether integration of dual-axis risk assessment with targeted vascular restoration can reduce severe maternal and perinatal outcomes represents a critical next step. If validated, such an approach may not only refine pregnancy care but also illuminate the shared vascular biology linking preeclampsia with long-term cardiovascular risk.

## Figures and Tables

**Table 2:** Comparative therapeutic modalities targeting angiogenic and vascular pathways in preeclampsia.

Characteristic	Oral Small-Molecule Pathway Modulator	siRNA/Oligonucleotide Targeting sFlt-1	Recombinant Biologic Protein Therapy
Primary mechanistic level	Upstream modulation of cytoprotective signalling with downstream reduction in anti-angiogenic drive	Direct suppression of sFlt-1 production at mRNA level	Augmentation of endogenous vasoactive or endothelial-protective protein pathways
Target specificity	Pathway-level modulation affecting multiple redox and angiogenic regulators	Highly specific transcript-level inhibition of FLT1	Protein-level receptor or enzymatic pathway activation
Route of administration	Oral	Parenteral (typically subcutaneous or intravenous)	Parenteral (intravenous or subcutaneous)
Pharmacokinetics	Small molecule distribution; potential for sustained systemic exposure	Dependent on delivery vehicle (e.g. lipid nanoparticle); transient effect; repeat dosing required	Protein half-life dependent; may require repeated monitored dosing
Placental transfer considerations	Molecular weight dependent; requires evaluation	Limited placental transfer expected but requires confirmation	Large protein structure; placental transfer generally low but requires evaluation
Manufacturing complexity	Chemical synthesis; scalable small molecule production	Complex oligonucleotide synthesis and nanoparticle formulation	Recombinant protein production, purification, and cold-chain stability
Iron/redox interface	Directly influences redox buffering and iron handling via HO-1 induction and antioxidant pathways	Indirect; reduces angiogenic stress but does not directly regulate iron metabolism	Mechanism dependent; may improve endothelial function without directly modifying iron homeostasis
Effect on ferroptotic susceptibility	Potential modulation via enhanced antioxidant buffering and reduced labile iron stress	Reduction in upstream angiogenic stress; ferroptosis effects indirect	Primarily vascular signalling effects; ferroptosis impact unclear
Clinical positioning	Suitable for prevention or early-intervention strategies in targeted high-risk populations	Potentially suited for treatment of severe cases in hospital	Likely to be suited for stabilisation in established disease
Health-system deployment considerations	Potential outpatient administration	Requires monitored administration and specialised handling	Typically requires supervised administration and logistics infrastructure
Integration with risk stratification tools	Compatible with longitudinal preventive use in biomarker-defined populations	Likely requires biomarker enrichment to optimise responder identification	May benefit from diagnostic enrichment to demonstrate rapid clinical impact

## Data Availability

No new data were created or analyzed in this study.
